# Tetrahydrocurcumin Upregulates the Adiponectin-AdipoR Pathway and Improves Insulin Signaling and Pancreatic β-Cell Function in High-Fat Diet/Streptozotocin-Induced Diabetic Obese Mice

**DOI:** 10.3390/nu13124552

**Published:** 2021-12-19

**Authors:** Yi-Zhen Tsai, Mei-Ling Tsai, Li-Yin Hsu, Chi-Tang Ho, Ching-Shu Lai

**Affiliations:** 1 Department of Seafood Science, National Kaohsiung University of Science and Technology, Kaohsiung 811, Taiwan; tsai1021538@gmail.com (Y.-Z.T.); ginger811129@gmail.com (L.-Y.H.); 2 Department of Food Science, Rutgers University, New Brunswick, NJ 08901, USA; mltsai@nkust.edu.tw (M.-L.T.); ctho@sebs.rutgers.edu (C.-T.H.)

**Keywords:** diabetes, obesity, adipose tissue, tetrahydrocurcumin, adiponectin, AdipoR, APPL1, β cell

## Abstract

Impairment of adiponectin production and function is closely associated with insulin resistance and type 2 diabetes, which are linked to obesity. Studies in animal models have documented the anti-diabetic effects of tetrahydrocurcumin (THC). Although several possible mechanisms have been proposed, the contribution of adiponectin signaling on THC-mediated antihyperglycemic effects remains unknown. Here, we report that adiposity, steatosis, and hyperglycemia were potently attenuated in high-fat diet/streptozotocin-induced diabetic obese mice after they received 20 and 100 mg/kg THC for 14 weeks. THC upregulated UCP-1 in adipose tissue and elevated adiponectin levels in the circulation. THC upregulated the AdipoR1/R2-APPL1-mediated pathway in the liver and skeletal muscle, which contributes to improved insulin signaling, glucose utilization, and lipid metabolism. Furthermore, THC treatment significantly (*p* < 0.05) preserved islet mass, reduced apoptosis, and restored defective insulin expression in the pancreatic β-cells of diabetic obese mice, which was accompanied by an elevation of AdipoR1 and APPL1. These results demonstrated a potential mechanism underlying the beneficial effects of THC against hyperglycemia via the adiponectin-AdipoR pathway, and thus, may lead to a novel therapeutic use for type 2 diabetes.

## 1. Introduction

Type 2 diabetes (T2D) is the most common type of diabetes in adults and frequently occurs in youth. It is also reported youth-onset T2D progresses more aggressively to complications than type 1 diabetes (T1D) [1]. Both type 1 diabetes (T1D) and T2D are characterized by hyperglycemia due to insulin deficiency; however, they have different pathogenic mechanisms. T1D results from T cell-mediated destruction of pancreatic β-cells, whereas insulin resistance is recognized as one of the critical mechanisms in the development of T2D [2]. Overweight and obesity, the consequences of excess nutrition, causes increased lipid circulation and inflammatory cytokines derived from dysregulated adipocytes. This also impairs the insulin sensitivity of the liver, muscle, adipose tissue, and other organs, resulting in a decrease in insulin-stimulated glucose uptake [3]. The compensatory increase in insulin secretion (hyperinsulinemia) by pancreatic β-cells overcome hyperglycemia accelerates their progressive decline, leading to insulin deficiency and eventually the onset of T2D [4]. Insulin resistance affects metabolic cross-talk between organs and is a central event in the pathogenesis of various metabolic diseases [3,4]. In addition, chronic hyperglycemia-driven microvascular and macrovascular complications facilitate organ dysfunction, disease development, and insulin resistance in T2D [4].

The pathological role of obesity in T2D is widely recognized. Lifestyle modification and weight-loss interventions are considered ideal and successful treatments for obesity-linked T2D [4]. However, several patients fail to achieve their goals or maintain them long-term. Current therapies for the treatment of T2D are focused on normalizing blood glucose either by increasing insulin secretion in existing pancreatic β-cells or by improving insulin sensitivity and reducing complications [5]. However, the complexity and variability of T2D in individual patients affect responses to therapies and fail to prevent hyperglycemia-related complications [6]. Potential side effects, such as hypoglycemia, weight gain, heart failure, pancreatitis, and unknown long-term safety also occur for currently available T2D pharmacotherapies [5]. Given that T2D is a complex and multifactorial disease, a therapeutic approach acting on multiple pathogenic mechanisms would be more beneficial for the management of disease progression.

Tetrahydrocurcumin (THC) is a major reductive metabolite of curcumin [7,8,9]. In addition, THC naturally occurs in the roots of *Zingiber mioga*, Z. officinale, and *Curcuma zedoaria* [10,11]. The potential biological properties of THC have been documented in numerous in vitro and in vivo studies; these effects include antioxidant, anti-inflammatory, anti-cancer, anti-aging, hepatoprotective, renal-protective, neuron-protective, and hypolipidemic activity. Thus THC exhibits several beneficial effects against human diseases [12,13]. The potential effects of THC on diabetes [14,15] and diabetic complication [16] have also been reported. Several studies have shown the hypoglycemic activity of THC, which might potentiate insulin secretion and decrease hepatic gluconeogenic enzyme activities in diabetic animals [17,18]. A recent study reported that THC increased insulin secretion via upregulation of glucagon-like peptide 1 (GLP-1) expression in the pancreas of *db*/*db* mice [19]. Our previous study demonstrated that THC ameliorated hyperglycemia, hyperinsulinemia, and insulin resistance in high-fat diet (HFD)-fed obese mice [20]. THC reduced impairment of hepatic insulin signaling and glucose metabolism in oleic acid-treated HepG2 cells and HFD-fed obese mice [20,21]. It is known adipocytes-derived endocrine hormones adipokines, such as adiponectin and leptin, contribute to systemic energy and metabolic homeostasis [22,23]. Information regarding the anti-hyperglycemic effect of THC, linking adipose tissue to other organs, is limited. The aim of this study was to investigate the potential role of THC in glucose homeostasis via adiponectin signaling to improve glucose and lipid metabolism, insulin resistance, and β-cell function in HFD/streptozotocin (STZ)-induced T2D mice along with the possible mechanisms involved in glucose homeostasis.

## 2. Materials and Methods

### 2.1. Chemicals and Reagents

THC (purity > 95%) was provided by American Medical Holding, Inc. (New York, NY, USA). The primary antibodies adiponectin, APPL1, ACC, p-ACC (Ser79), p-Akt (Ser473), Akt, p-AMPKα (Thr172), AMPKα, FAS, p-GS (Ser641), glucagon, PCNA, and UCP-1 were from Cell Signaling Technology (Beverly, MA, USA). F4/80, AdipoR2, IRβ, E-cadherin, PPARα, CPT-1, Glut2, Glut4, and insulin antibodies was purchased from Santa Cruz Biotechnology (Santa Cruz, CA, USA). AdipoR1, CD11b, CD163, and PDX-1 antibodies were from Abcam (Cambridge, UK). β-Actin and p-IRβ (Tyr1158/Tyr1162/Tyr1163) antibodies were purchased from Sigma Chemical Co. (St. Louis, MO, USA). GAPDH and Caspase 3 antibody was obtained from GeneTex Inc. (Irvine, CA, USA) and IMGENEX (San Diego, CA, USA), respectively. The HRP-conjugated secondary antibodies were from Jackson ImmunoResearch (West Grove, PA, USA). STZ and other chemicals was purchased from Sigma Aldrich (St. Louis, MO, USA).

### 2.2. Animal and Treatments

All animal care and experiments were carried out in accordance with the Guide for the Care and Use of Laboratory Animals of the National Institutes of Health. The experimental protocol was approved by the Institutional Animal Care and Use Committee of the National Kaohsiung University of Science and Technology (IACUC, NKUST, 0107-AAAP-006). Four-week-old male C57BL/6J mice were purchased from the BioLASCO Experimental Animal Center (Taiwan Co., Ltd., Taipei, Taiwan). All mice had free access to food and water and were maintained under a daily 12 h light/12 h dark cycle at 25 ± 1 °C. After one-week of acclimation, a total of 24 mice were fed a HFD from 4 weeks of age and the other with a normal diet (ND). After a 6-week HFD-fed, all obese mice were intraperitoneal injection with 35 mg/kg STZ for 5 consecutive days and other mice fed ND were received citrate buffer alone. The fasting blood glucose in HFD-fed obese mice was measured at week 8. The diabetic obese mice with fasting blood glucose more than 200 mg/dl were randomly distributed into three groups: an HFD/STZ alone group, 20 mg/kg and 100 mg/kg THC group, in which the mice were continued on a HFD and orally administration with corn oil, 20 and 100 mg/kg body weight of THC per day for 14 weeks, respectively. The dose of 20 and 100 mg/kg in mice is equivalent to 1.6 and 8.1 mg/kg in humans, respectively, according to the formula based on body surface area [24]. Mice fed ND were also divided into two groups: a ND alone group and THC alone group, in which the mice were continued fed with a ND and orally administration with corn oil and 100 mg/kg THC throughout the study. Body weight and food intake were measured weekly. At the end of the study, blood, adipose tissues, liver, spleen, kidney, and pancreas were collected for further analysis.

### 2.3. Oral Glucose Tolerance Test, Serum Parameters, and Hepatic Triacylglycerol

The oral glucose tolerance test (OGTT) was performed at week 20 (after THC treatment for 12 weeks). Mice were fasted for 6 h (morning fast) and orally gavage 1 g/kg glucose. Blood glucose was measured by a OneTouch ultra blood glucose meter (LifeScan, Inc., Milpitas, CA, USA) at 0, 30, 60, 90, and 120 min after administration. The areas under the curve (AUC) of blood glucose levels in each groups were calculated. Fasting serum levels of GOT, GPT, TG, TCHO, and cholesterol/HDL-C were determined using a blood biochemistry analyzer (Fujifilm Dri-Chem 3500s; Fujifilm). Serum insulin (Mercodia Mouse Insulin Elisa, Uppsala, Sweden) was measured by using commercially available ELISA kit. For detection of serum adiponectin (full-length), the serum samples were diluted (1:1000) and assayed by a Mouse Adiponectin ELISA kit according to the manufacturer’s instructions (Millipore; Temecula, CA, USA). The dynamic range of the Mouse Adiponectin ELISA kit is 1–50 ng/mL, and its sensitivity is 0.2 ng/mL. Hepatic TG content was measured by using a Triglyceride Colorimetric Assay Kit (Cayman, Ann Arbor, MI, USA), and the results were expressed as mg TG per gram of liver tissue.

### 2.4. Metabolic Measurement

Indirect calorimetry was performed at week 16 (after THC treatment for 8 weeks) using TSE metabolic chambers (TSE Labmaster System, Bad Homburg, Germany). Mice were individually housed in the metabolic cages and acclimatized for 24 h prior to the beginning of an additional 24 h of hourly recording of metabolic parameters. The parameters include food consumption, water intake, the volume of O_2_ (VO_2_), the volume of CO_2_ (VCO_2_), respiratory exchange ratio (RER = VO_2_/VCO_2_) and heat production (kcal/h) were recorded and calculated by TSE software.

### 2.5. Histological, Immunohistochemical and Immunofluorescence Analysis

Epididymal fat tissue, liver tissue and pancreatic tissue were collected and immediately fixed in 10% buffered formalin, embedded in paraffin and cut into 4–5 μm sections. The histology was examined after hematoxylin and eosin (H&E) staining by a light microscopy (Olympus, Tokyo, Japan). Images of adipose tissue section were captured in 10 random fields per section (200× magnification) to measure adipocyte size. For islet mass analysis, islet area of each section was measured using ImageJ software and divided by the total area of pancreas area, and expressed as the percentage of islet area relative to the total pancreatic area. The islet mass was calculated as the islet area divided by the total area of pancreas area. For immunohistochemical staining, the sections were processed as described previously and subsequently incubated with primary antibodies (CD11b, CD163, insulin, glucagon, PDX-1, Glut2, and APPL1) for 1 h at room temperature. After washing, the signal was visualized with a Mouse/Rabbit PolyDetector Plus HRP/DAB System (BioSB, Santa Barbara, CA, USA). For immunofluorescence staining, the sections were incubated with primary antibodies (F4/80, adiponectin, Glut2, Glut4, and AdipoR1) for 1 h, followed by incubation with the corresponding Dylight 488-goat anti-Rabbit IgG and Rhodamine (TRITC)-AffiniPure goat anti-mouse IgG secondary antibodies (Jackson ImmunoResearch, West Grove, PA, USA). The nuclei were counterstained with 4′, 6-diamidino-2-phenylindole (DAPI) (Sigma-Aldrich Co. St. Louis, MO, USA). Insulin/caspase-3 and Insulin/PCNA double immunofluorescence staining was performed to measure β-cell apoptosis and proliferation.

### 2.6. Western Blot Analysis

Briefly, total protein of liver and muscle tissue was extracted and 50 μg of protein were separated by SDS−polyacrylamide gel electrophoresis. The gels were then transferred to PVDF membranes and incubated with 20 mM Tris-HCl buffer containing 1% BSA at room temperature for 1 h. The blots were further probed with primary antibodies overnight at 4 °C and then incubated with HRP-conjugated secondary antibodies at room temperature for 1 h. After washing step, the transferred proteins were detected by an enhanced chemiluminescence detection kit (ECL; Amersham Pharmacia Biotech, Buckinghamshire, UK) and exposed to X-ray Film. The protein density was quantified using ImageJ software.

### 2.7. Reverse Transcription PCR

Total RNA was extracted from liver tissue using Trizol^®^ Reagent according to the manufacturer’s instructions (Invitrogen, Life Technologies, Carlsbad, CA, USA), and then 2 μg was transcribed into cDNA using SuperScript II Reverse Transcriptase (Invitrogen, Renfrewshire, UK). The PCR reactions were performed in an Applied Biosystems 2720 Thermal Cycler with Cpt-1a, Pepck1 and β-actin primers as previously described [20]. The PCR products were then assessed by gel electrophoresis and visualized by ethidium bromide staining.

### 2.8. Statistical Analysis

Data were presented as means ± SE for the indicated number of independently performed experiments. Comparisons of statistical significance between groups were analyzed by one-way analysis of variance (ANOVA) followed by a Tukey’s post hoc multiple comparison test. A *p* value < 0.05 was considered statistically significant. Analysis was performed using SAS v 9.1 software.

## 3. Results

### 3.1. THC Ameliorates Weight Gain, Adiposity, and Dyslipidemia in Diabetic Obese Mice

Supplementary Figure S1 shows body weight changes among the groups during the experiment. Body weight gradually increased in mice fed a HFD for the first 6 weeks compared to that in the ND group. A lower body weight was found after STZ administration in the HFD-fed obese mice (week 7); however, this was not the case in the ND groups. The diabetic obese mice were orally treated with THC for an additional 14 weeks with a continued HFD, and the weight gain is shown in Table 1. The initial body weight (week 8) among groups was similar among HFD-fed groups. THC treatment at 20 and 100 mg/kg significantly (*p* < 0.05) attenuated the final body weight and weight gain in diabetic obese mice. However, a decrease in food intake occurred with 100 mg/kg THC treatment, reflecting that the reduced weight gain may have been partly due to reduced food intake.

Supplementary Figure S2A illustrates that relative epididymal, perigonadal, and mesenteric weights were approximately 50% smaller in THC-treated groups than in HFD/STZ alone mice. Correspondingly, we found that THC caused a marked decrease in the average adipocyte size (Supplementary Figure S2C,D). THC had no effect on the relative brown adipose tissue (BAT) weight compared to that in the HFD/STZ alone group (Supplementary Figure S2B). No significant difference was noted in the relative liver, kidney, and pancreas weights among any of the HFD/STZ groups; however, it was observed that the relative spleen weight increased in 20 and 100 mg/kg THC group compared to that in the HFD/STZ alone group. In addition, mice in the HFD/STZ alone group showed notably higher serum levels of TG and TCHO compared to those in the ND-fed alone group, and these levels were significantly (*p* < 0.05) reduced by THC treatment. Altogether, these results demonstrated that 20 mg/kg THC treatment ameliorated obesity and improved dyslipidemia in HFD/STZ-induced diabetic obese mice, whereas 100 mg/kg THC seems to exert a positive effect by reducing food intake.

### 3.2. THC Relieves Hyperglycemia, Hyperinsulinemia, Improves Glucose Tolerance, and Energy Metabolism in Diabetic Obese Mice

**Figure 1:**
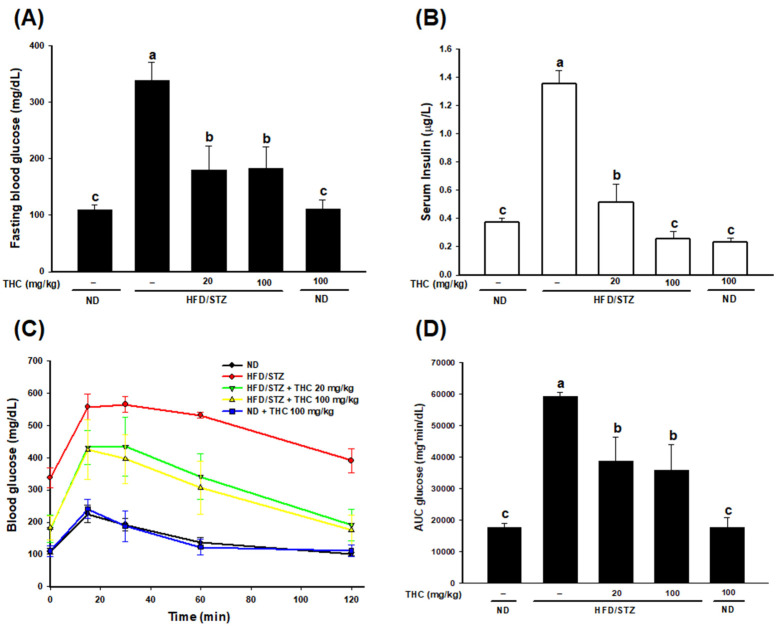
THC intervention reduces HFD/STZ-induced impaired glucose homeostasis and hyperinsulinemia. (**A**) Fasting blood glucose. (**B**) Fasting serum insulin levels. (**C**) Blood glucose during an OGTT and (**D**) corresponding AUC. Data are presented as mean ± SEM (*n* = 5–6). Statistical differences (*p* < 0.05) between groups were evaluated by one-way ANOVA with Tukey’s post-hoc test and were labeled as different letters (a, b, and c). AUC: areas under the curve; HFD/STZ: high fat diet/ streptozotocin; ND: normal diet; OGTT, oral glucose tolerance test; THC: tetrahydrocurcumin.

Compared to the HFD/STZ alone group, 20 mg/kg THC administration notably attenuated hyperglycemia and hyperinsulinemia in diabetic obese mice (Figure 1A,B). As shown in Figure 1C, blood glucose levels and the corresponding AUC profile were observed to be significantly (*p* < 0.05) lower with THC treatment at all indicated time points compared to those in the HFD/STZ alone group. The corresponding AUC profile also reflected a significant (*p* < 0.05) decrease in blood glucose in mice with THC treatment compared to that in the HFD/STZ alone group (Figure 1D). These results indicated that THC improved hyperglycemia, hyperinsulinemia, and impaired glucose tolerance in HFD/STZ-induced diabetic obese mice.

**Figure 2:**
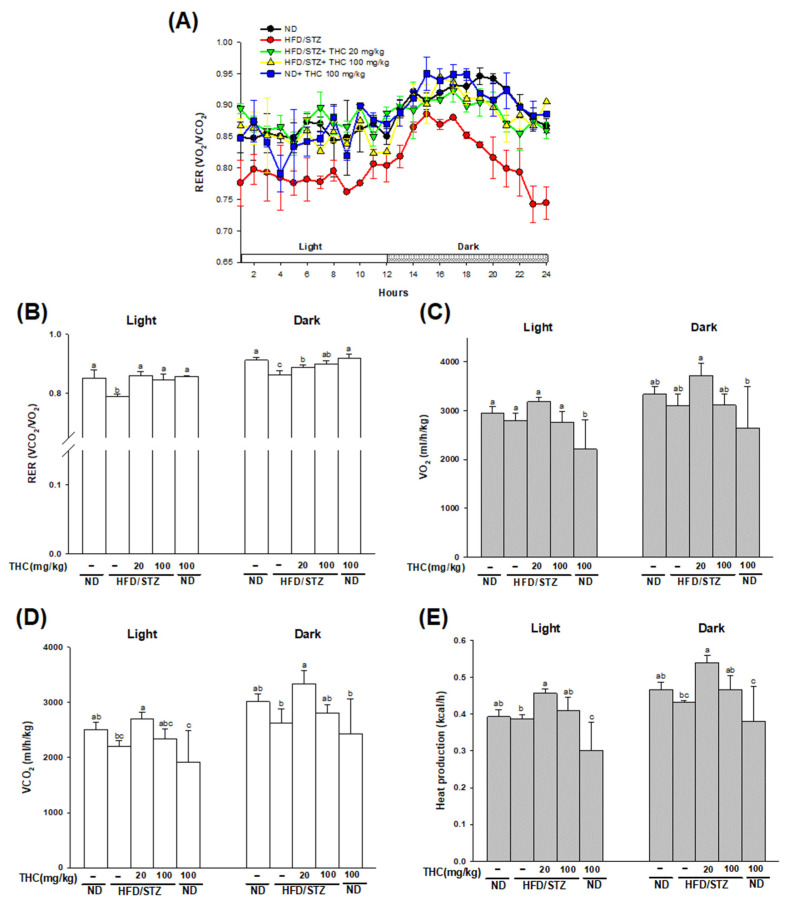
Effect of THC intervention on whole-body energy metabolism in HFD/STZ treated mice. Indirect calorimetry was performed on the TSE system. (**A**) Respiratory exchange ratios (RER = VCO_2_/VO_2_) during a 24h period. (**B**) Average RER, (**C**) O_2_ consumption, (**D**) CO_2_ production and (**E**) heat production during the light and dark phase was calculated. Data are presented as mean ± SEM (*n* = 4–5). Statistical differences (*p* < 0.05) between groups were evaluated by one-way ANOVA with Tukey’s post-hoc test and were labeled as different letters (a, b, and c).

Figure 2 shows the results of the indirect calorimetry. The RER of the ND alone and HFD/STZ alone group in the light phase was ≈0.85 and 0.79, respectively, indicating that the substrate type utilized in the two groups was different. The RER in THC-treated mice was similar to that in the ND alone group in the light phase, implying that THC restored the utilization of carbohydrates and fat as fuel substrates in diabetic obese mice (Figure 2B). The pattern of RER among the groups in the dark phase was similar to that in the light phase. The VO_2_, VCO_2_ and heat production was significantly (*p* < 0.05) higher in 20 mg/kg THC-fed mice than in the HFD/STZ alone group (Figure 2C–E). A marginal yet not significant increase in VCO_2_ and heat production was observed in mice treated with 100 mg/kg THC compared to that in mice in the HFD/STZ alone group. Therefore, we suggest that THC at 20 mg/kg reduced adiposity with higher energy expenditure via an increase in heat production. However, we cannot rule out that physical activity may also play a role in THC-mediated increased heat production.

### 3.3. THC Alleviates Adipose Chronic Inflammation, Improves Adiponectin Secretion and Glut4 Levels in Diabetic Obese Mice

We found that macrophage infiltration (F4/80) and M1 macrophages expressing the CD11b marker notably accumulated in the epididymal adipose tissue of the HFD/STZ alone group, accompanied by a decrease in M2 macrophages with the CD163 marker, when compared to ND-fed mice (Supplementary Figure S3A,B). In contrast, THC significantly (*p* < 0.05) and dramatically diminished macrophage infiltration and modulated the M1/M2 ratio in epididymal adipose tissue of diabetic obese mice. 

**Figure 3:**
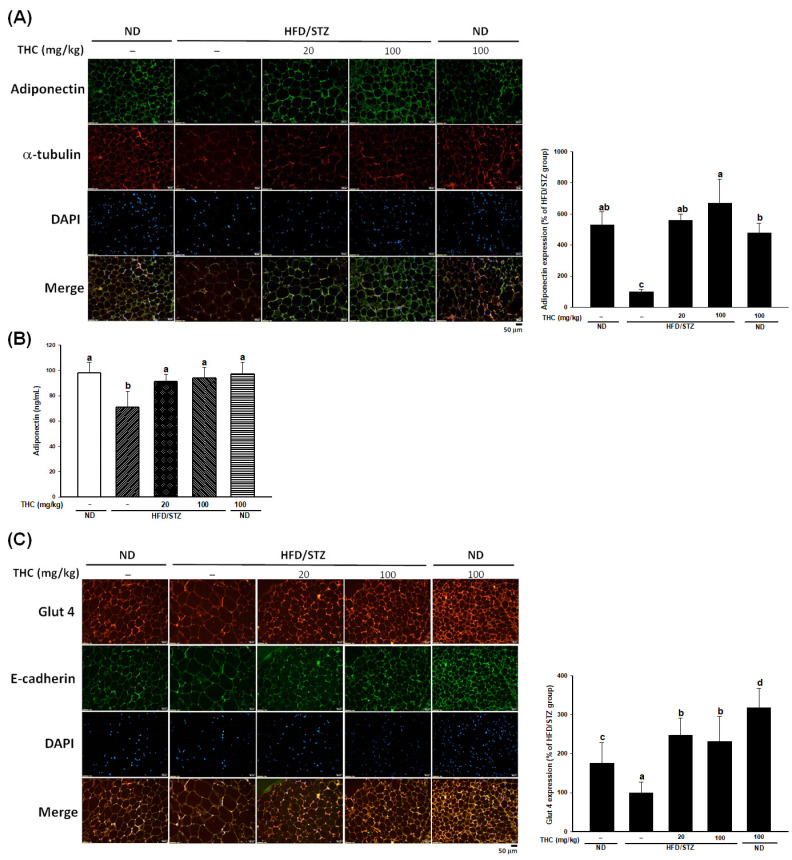
THC intervention improves adiponectin in HFD/STZ treated mice. (**A**) Immunofluorescence staining for adiponectin (green), α-tubulin (red), and DAPI (blue) of epididymal adipose tissue. (**B**) The plasma adiponectin level of each group was measured at the end of study. (**C**) Representative images of immunofluorescence staining for Glut4 (red) and E-cadherin (green) in epididymal adipose tissue. The quantitative result of immunostaining was conducted by ImageJ. Data are presented as mean ± SEM (*n* = 5–6). Statistical differences (*p* < 0.05) between groups were evaluated by one-way ANOVA with Tukey’s post-hoc test and were labeled as different letters (a, b, and c).

Adiponectin levels in adipose tissue and serum were significantly reduced (*p* < 0.05) in the HFD/STZ alone group (Figure 3A,B), along with the decreased Glut 4 levels in the plasma membrane of adipose tissue (Figure 3C) compared to those in the ND alone group. THC treatment effectively restored adipose and serum adiponectin levels. Moreover, the decrease in Glut4 levels were significantly and effectively elevated by THC treatment at both dosages. Moreover, upregulation of uncoupling protein-1(UCP-1) in epididymal adipose tissue of diabetic obese mice was observed following THC treatment (Supplementary Figure S4). This implies that THC may promote WAT browning, which contributes to energy expenditure enhancement by promoting heat production in diabetic obese mice. It is important to note that, we do not exclude the possibility that an increase in physical activity may also contribute to the heat production in THC-treated diabetic obese mice. Future work is required to clarify this issue.

### 3.4. THC Ameliorates Steatosis, Insulin Signaling and Upregulates AdipoR-APPL1 Signaling in the Liver and Muscle of Diabetic Obese Mice

**Figure 4:**
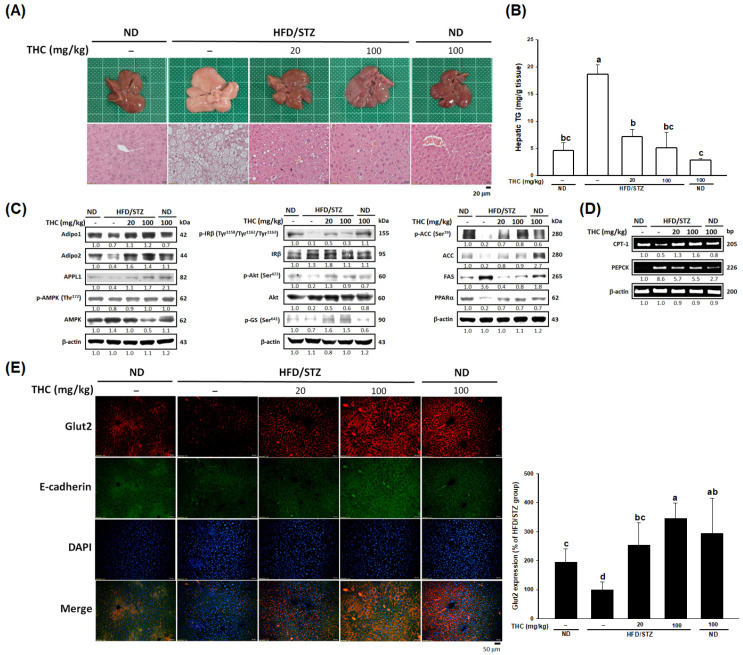
Amelioration of hepatic steatosis and by THC in HFD/STZ treated mice. (**A**) Macroscopic appearance (upper) and H&E staining (lower) of liver tissue. (**B**) Serum level of hepatic TG content was measured in each group (*n* = 6). (**C**) AdipoR-APPL1 signaling, insulin signaling, lipogenesis and PPARα protein level in the liver was detected by Western blot analysis. (**D**) Hepatic mRNA levels of CPT-1 and PEPCK was analyzed by RT-PCR. The data of Western blot and PCR shown are representative of three independent experiments with similar results. (**E**) Immunofluorescence staining with hepatic Glut2 (red), E-cadherin (green), and DAPI (blue). Quantitative data of immunostaining was performed by ImageJ (*n* = 5). Data are presented as mean ± SEM (*n* = 5–6). Statistical differences (*p* < 0.05) between groups were evaluated by one-way ANOVA with Tukey’s post-hoc test and were labeled as different letters (a, b, and c).

Given that THC upregulated adiponectin in both adipose tissue and serum of diabetic obese mice, we aimed to investigate whether this effect contributed to the improvement of lipid and glucose metabolism in the liver and other tissues. Figure 3A illustrates THC treatment reduced a greater number of ballooned hepatocytes and hepatic steatosis compared to that of HFD/STZ alone group, with the decreased serum GOT, GPT (Table 1), and hepatic TG levels (Figure 4B). Western blot analysis showed decreased protein levels of adiponectin receptor 1 (AdipoR1), adiponectin receptor 2 (AdipoR2), and APPL1 in diabetic obese mice were reversed following THC administration (Figure 4C, left). Moreover, the impaired insulin signaling, tyrosine phosphorylation of insulin receptor β (IRβ) and downstream phosphorylation of Akt, was largely ameliorated by THC administration in diabetic obese mice (Figure 4C, middle). 

We also examined the effect of THC on lipid and glucose metabolism in diabetic obese mice. As shown in Figure 4C (right), THC administration significantly (*p* < 0.05) downregulated the phosphorylation of ACC and FAS protein levels, and upregulated that of PPARα (Figure 3C, right) and CPT-1 (Figure 4D, upper), indicating that THC might reduce hepatic lipogenesis and induce β-oxidation. In addition, inactivation of glycogen synthase (GS) (Figure 4C, middle) and increased gene expression of PEPCK (Figure 4D, lower) was improved by THC. The reduced Glut2 levels in the livers of diabetic obese mice were significantly elevated following THC treatment (Figure 4E). A similar effect was observed in muscle (Supplementary Figure S5). Collectively, these observations suggest that THC-ameliorated steatosis might contribute to the upregulation of AdipoR1-AMPK signaling and the AdipoR2-PPARα pathway, thus improving lipid and glucose metabolism in the liver and muscle of HFD/STZ-induced diabetic obese mice.

### 3.5. THC Restores β-Cell Function in Diabetic Obese Mice

**Figure 5:**
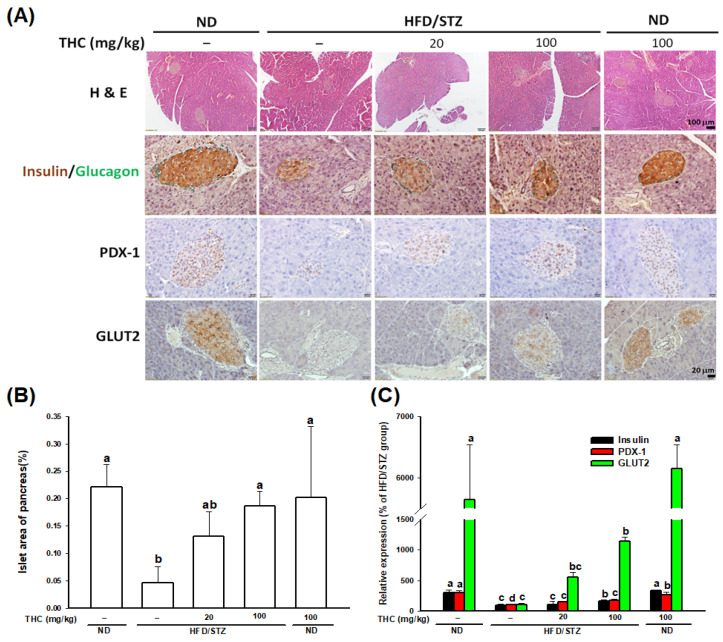
Effect of THC administration on pancreatic morphology, islet mass and function in HFD/STZ treated mice. (**A**) Representative images of pancreas histology by H&E staining. Islet morphology and β-cell function was examined by immunostaining with insulin (β cells), glucagon (α cells), PDX-1, and Glut2. (**B**) Islet area was quantified by the ImageJ and normalized to pancreatic section area. Data are expressed as mean ± SEM (*n* = 5–6). (**C**) Quantification of relative insulin, PDX-1, and Glut 2 expression was performed by ImageJ. Statistical differences (*p* < 0.05) between groups were evaluated by one-way ANOVA with Tukey’s post-hoc test and were labeled as different letters (a, b, and c).

**Figure 6:**
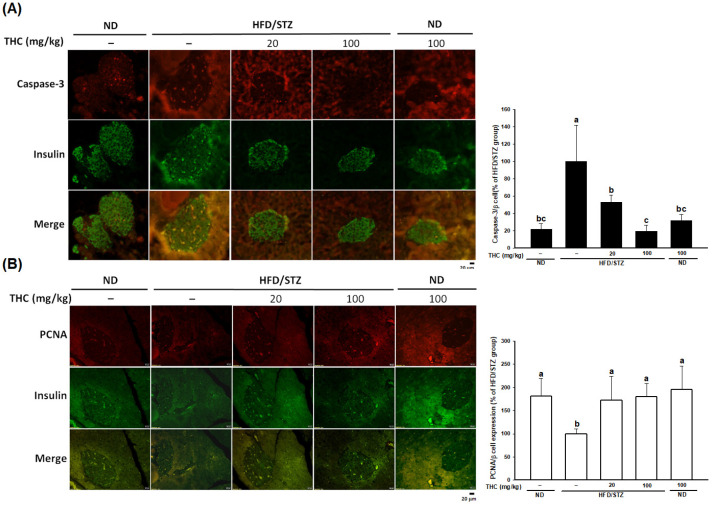
Effect of THC on β-cell apoptosis and proliferation in HFD/STZ treated mice. (**A**) Apoptosis of β-cell was analyzed by double immunofluorescence staining with caspase-3 (red) and insulin (green). (**B**) Proliferative β-cells were analyzed by double immunofluorescence staining with PCNA (red) and insulin (green). Quantification was performed by ImageJ. Data are expressed as mean ± SEM (*n* = 6). Statistical differences (*p* < 0.05) between groups were evaluated by one-way ANOVA with Tukey’s post-hoc test and were labeled as different letters (a, b, and c).

The relative pancreas weight was similar among HFD/STZ alone and THC groups (Table 1). H&E staining of pancreatic sections showed that islet numbers and mass were reduced in the HFD/STZ alone group (Figure 5A,B). In addition, islet morphology in the HFD/STZ alone group showed uneven boundaries and the architecture was deregulated, with disruption of α-cells (glucagon) and β-cells (insulin) (Figure 5A). In contrast, diabetic obese mice treated with THC displayed an increased number and enlargement of islets, and improved islet architecture. Thereafter, we examined the effect of THC on the modulation of pancreatic β-cell fate and function in HFD/STZ-induced diabetic obese mice. Figure 5A showed a marked decrease in insulin intensity in pancreatic β-cells in mice treated with HFD/STZ alone. This decrease was significantly (*p* < 0.05) improved by 100 mg/kg THC treatment (Figure 5A,C); however, this may be partly due to a lower food intake. The attenuated PDX-1 and its downstream target, Glut2, in pancreatic β-cells of diabetic obese mice were also restored following THC treatment (Figure 5A,C). We also found a marked decrease in caspase-3 staining of pancreatic β-cells in the diabetic obese mice following by THC administration (Figure 6A). In addition, pancreatic β-cells in THC-treated mice showed significantly (*p* < 0.05) higher PCNA staining compared to that in HFD/STZ alone group (Figure 6B). These results suggested that THC reduced apoptosis, increased proliferation of pancreatic β-cells, and restored their function in diabetic obese mice.

### 3.6. THC Resorted AdipoR1 Expression in Diabetic Obese Mice

**Figure 7:**
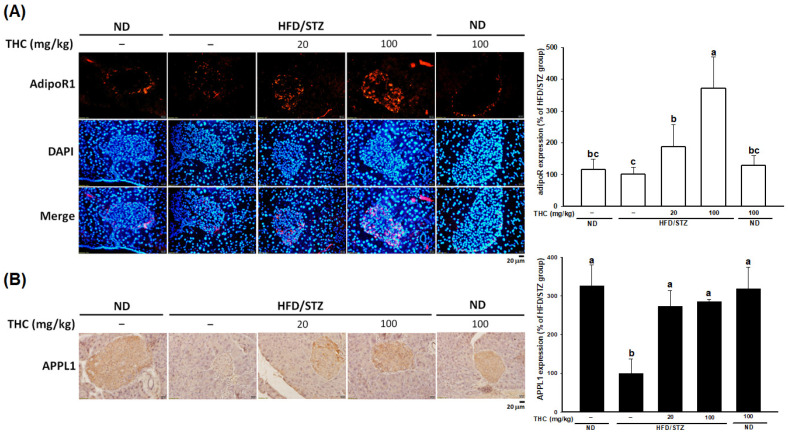
Upregulation of AdipoR1 and APPL1 in pancreatic islets by THC in HFD/STZ treated mice. (**A**) Immunofluorescence staining of AdipoR1 (red) and DAPI (blue) in pancreas sections. (**B**) APPL1 expression was detected in pancreas sections by immunohistochemical analysis. Quantification of AdipoR1 and APPL1 was performed by ImageJ. Data are expressed as mean ± SEM (*n* = 5–6). Statistical differences (*p* < 0.05) between groups were evaluated by one-way ANOVA with Tukey’s post-hoc test and were labeled as different letters (a, b, and c).

To investigate whether THC restores adiponectin-mediated function in the pancreas in diabetes, we examined AdipoR1 and APPL1 levels with immunofluorescence staining. As shown in Figure 7A, the expression pattern of AdipoR1 in islet cells between the ND alone and HFD/STZ alone groups was similar. However, THC treatment dramatically upregulated AdipoR1 in pancreatic islets at both doses. In accordance with increased AdipoR1, pronounced expression of APPL1 in pancreatic islets was also observed following THC administration (Figure 7B). We showed that circulating adiponectin markedly increased following THC administration (Figure 3B) in diabetic obese mice, indicating that THC upregulated serum adiponectin, and pancreatic AdipoR1 and APPL1 may contribute to restoring β-cell loss and dysfunction in diabetic obese mice.

## 4. Discussion

In this study, we demonstrated that THC restored the endocrine function of adipose tissue, as evidenced by the recovery of adiponectin expression and secretion. THC-mediated the restoration of circulating adiponectin and the recovery of adiponectin signaling in the liver, muscle, and pancreas via upregulated AdipoR1/R2 and adaptor protein, phosphotyrosine interacting with PH domain and leucine zipper 1 (APPL1). This exhibited beneficial effects on glucose or lipid metabolism, improved insulin resistance, improved *β*-cell function, and lowered hyperglycemia in HFD/STZ-induced diabetic obese mice. To the best of our knowledge, our study is the first to show that THC increases circulating adiponectin accompanied by upregulated AdipoR1/R2-APPL1 expression, which may contribute to an anti-diabetic effect.

Although curcumin is recognized as an active ingredient of turmeric and exhibits many beneficial effects for human diseases, its potential for nutraceutical and pharmaceutical applications is limited by its poor bioavailability [25]. The chemical instability, low absorption, extensive metabolism, and rapid elimination contributes to the poor bioavailability of curcumin [25,26]. In contrast, evidence shows THC displays better bioavailability than curcumin. Male ddY mice fed a diet containing 0.5% curcumin or THC for 4 weeks, only a small amount of free curcumin and its conjugates (sulfates and glucuronides) was detected in the serum (0.6 ± 0.1 μmol/L), and it was not found in the liver. However, a larger amount of free tetrahydrocurcumin, and its conjugates were detected in the serum (43.4 ± 15.5 μmol/L for free and conjugates) and liver (2.5 ± 0.6 μmol/L for free and 7.9 ± 1.6 μmol/L for conjugates) [27]. Another study demonstrated mice fed a diet with 0.05% (~83 mg/kg body weight) curcumin or THC for 16 weeks showed a higher plasma level of THC (0.270 ± 0.003 μg/L) than that of curcumin (0.035 ± 0.014 μg/L) [28]. This information reveals that THC may be more easily absorbed through the gastrointestinal tract than curcumin to exhibit its better bioavailability. Apart from this, the better solubility, chemical, and metabolic stability also contributes to the higher bioavailability of THC than curcumin [29,30]. In addition to the better bioavailability, THC demonstrates a superior antioxidative activity and less cytotoxicity than curcumin. THC is reported to exhibit a stronger activity for radical scavenging, inhibition of lipid peroxidation, and reduction of ROS production than curcumin [31,32]. Curcumin acts as an antioxidant but also shows a prooxidant effect at higher concentrations [33,34]. The phenyl hydroxyl groups, phenyl methoxy groups, and α,β-unsaturated carbonyl moiety of curcumin that mediate ROS generation are contributing to its prooxidant effect [35]. In contrast to curcumin, THC is not pro-oxidative because of the absence of conjugated bonds in the central seven-carbon chain, which may contribute to its effect against ROS-induced cellular damage and death [32,36]. While numerous studies suggest the anti-obesity and anti-diabetic efficacy of curcumin, THC may still hold the biological potential owing to its better bioavailability, potent antioxidant activity, and less cytotoxic effect.

This study showed that administration of THC to HFD/STZ-induced diabetic obese mice for 14 weeks resulted in a reduction in weight gain and adipose mas. We found 20 mg/kg THC reduced body weight gain and adipose tissue weight in obese mice. The decreased expansion of adipocytes (hypertrophy) and suppression of adipocyte differentiation (hyperplasia) appear to be the primary mechanism by which 20 mg/kg THC reduces adipose mass in HFD-fed obese mice, which is reported in our previous study. While in the case of 100 mg/kg THC treatment, the reduced food intake may act as a major mechanism for the decreased adiposity in diabetic obese mice [20]. Increased energy expenditure is another possible mechanism underlying THC-mediated adiposity alleviation and metabolic homeostasis in diabetic obese mice, which was verified by an increase in heat production in the 20 mg/kg THC-treated group. Although THC administration did not restore the decreased weight of BAT in diabetic obese mice, the BAT marker, UCP-1, was upregulated in white adipose tissue (WAT) following THC treatment. In addition, our previous study showed THC increased lipolysis, upregulated PPARα and CPT-1 in oleic acid-induced steatosis HepG2 cells, which may stimulate fatty acid oxidation [21]. THC upregulated PPARα and CPT-1 in the liver and muscle of diabetic obese mice in this study was also found. This implies that THC may promote WAT browning and fatty acid oxidation, which contributes to energy expenditure enhancement by promoting heat production in diabetic obese mice. Although an increase in physical activity is an important contributor to energy expenditure whereas we did not measure this issue in our current study. THC-mediated metabolic effect in diabetic obese mice may also involve the upregulation of insulin signaling. We previously demonstrated that THC restored impaired insulin signaling glucose, and lipid metabolism in oleic acid-treated HepG2 cells and HFD-fed obese mice [20,21], which was also found in this study.

Adiponectin is a hormone exclusively derived from adipocytes and plays an important role in maintaining energy homeostasis [23]. Decreased adiponectin is implicated in the development of insulin resistance, obesity, and type 2 diabetes, whereas its elevation improves insulin sensitivity and energy metabolism [37]. This study demonstrated that THC was able to improve hyperglycemia and metabolic homeostasis in HFD/STZ-induced diabetic obese mice. The first potential mechanism may involve improved peripheral insulin sensitivity via adiponectin signaling following THC administration. We found a notable decrease in adiponectin levels in serum and epididymal adipose tissue of diabetic obese mice. In contrast, THC markedly upregulated adiponectin, with increased Glut4 in epididymal adipose tissue, which may ameliorate glucose uptake in WAT in diabetic obese mice. The biological functions of adiponectin are mediated via its two major receptors, AdipoR1 and AdipoR2, which are predominantly expressed in the skeletal muscles and liver, respectively [38]. AdipoR is known to interact with APPL1 in response to adiponectin, an adaptor protein, to activate AMPK signaling and the PPARα pathway, thus mediating glucose metabolism and lipid oxidation [39]. Apart from increased adiponectin, we showed that THC upregulated AdipoR1, AdipoR2, and APPL1, which activated downstream AMPK in the liver and skeletal muscle of diabetic obese mice. The upregulation of AdipoR1-AMPK signaling by THC increased insulin signaling (p-IRβ and p-Akt), further increased expression of glucose transporters (Glut 2 and Glut 4), and downregulation of lipogenic enzymes (ACC and FAS) in the liver and skeletal muscle of diabetic obese mice. It has been reported that crosstalk between adiponectin and insulin signaling may be coordinated in part by APPL1. APPL1 potentiates insulin-mediated inhibition of hepatic gluconeogenesis by promoting Akt activation [40]. APPL1 also promotes insulin-stimulated recruitment of insulin receptor substrate 1/2 to IRβ [41]. This study shows that THC improved phosphorylation of IRβ and downstream Akt in the liver and muscle, which was accompanied by the inactivation of glycogen synthase and increased PEPCK transcription. Based on these results, our study revealed that the mechanism underlying the role of THC in sensitizing insulin signaling may involve the upregulation of APPL1. In addition to these findings, THC induced the AdipoR2-PPARα pathway in both the liver and skeletal muscle, leading to upregulated CPT-1, which may increase fatty acid oxidation, although this was not examined here. Overall, the above effects accounted for the reduced hepatic TG content and improved glucose metabolism in the liver and skeletal muscle, which further supports the beneficial effect of THC on insulin resistance, hyperglycemia, steatosis, and whole-body metabolism in HFD/STZ-induced diabetic obese mice.

THC-mediated restoration of insulin upregulation is the second mechanism underlying the blood glucose-lowering effect in our current study. It was previously shown that THC significantly decreased blood glucose and increased serum level of insulin in diabetic animals [17,18]; however, the mechanism toward β-cells has not yet been elucidated despite reported THC-restored pancreatic histology in STZ/nicotinamide-induced diabetic Wistar rats [42]. Our current data clearly demonstrated that THC improved islet architecture and attenuated the loss of islet mass in diabetic obese mice by decreasing apoptosis and increasing β-cell proliferation. PDX-1 is a transcription factor that plays a central role in β-cell function and its disruption develops diabetes with impaired expression of insulin and Glut2 [43]. Our observations showed that 20 mg/kg THC upregulated pancreatic PDX-1 and its downstream Glut2 in diabetic obese mice. Although the insulin expression did not change, 20 mg/kg THC treatment had a tendency for higher pancreatic weight and islet area. On the other hand, 100 mg/kg THC administration caused a lower food intake, which may partially account for the favorable effect on defective β-cell function in diabetic obese mice. We further explored the role of adiponectin signaling in THC-mediated anti-diabetic effects and showed the upregulation of AdipoR1 and APPL1 in the pancreas of HFD/STZ-induced diabetic obese mice. Research has shown that AdipoR1 and AdipoR2 are expressed in pancreatic β-cells [44], and adiponectin protects β-cells against apoptosis and stimulates insulin secretion [45]. In pancreatic β-cells, the adapter protein APPL1 appears to be controlling insulin exocytosis via an Akt-dependent pathway [46]. Furthermore, APPL1 has a protective role in the pancreas by diminishing islet inflammation and β-cell apoptosis in STZ-induced type 1 diabetic mice [47]. According to these studies, the evidence from the present study demonstrated that THC protects β-cells against apoptosis and improves their function in HFD/STZ-induced diabetic obese mice, in part by upregulation of AdipoR1 and APPL1.

The first major challenge in our study was that the beneficial effect of 100 mg/kg THC treatment in diabetic obese mice, partly because the reduced food intake. In contrast, 20 mg/kg THC administration confers an improving effect for adiposity, hyperglycemia, and other metabolic dysfunctions in HFD/STZ-induced diabetic obese mice, even though with a significantly increased food intake. Pari and Murugan demonstrated that diabetic rats treated with 20, 40, and 80 mg/kg THC for 45 d showed a dose-dependent effect on fasting blood glucose and plasma insulin [18]. In our study, the difference in mechanisms by which 20 mg/kg and 100 mg/kg THC exert beneficial effects may explain why no apparent dose dependence. Moreover, different disease models and animal species used in these two studies may also accounts for the dose-independent effect observed in our study. Second, our results suggest that THC-mediated adiponectin and adiponectin receptors are associated with the improvement of glucose homeostasis in diabetic obese mice; however, their importance is not investigated for the beneficial effect of THC exhibited in this study and must be the focus of future studies. In addition, our study showed THC treatment caused unexpected splenomegaly in HFD/STZ-induced diabetic obese mice, which was not found in our previous study [20]. It is also reported that Wistar rats oral administration with THC at 100, 200, and 400 mg/kg showed no effect on absolute and relative spleen weight in a 90-day subchronic toxicity study [48]. We cannot address this issue so far and needs examination in the further study.

In conclusion, this work demonstrated that 20 mg/kg THC ameliorated hyperglycemia in HFD/STZ-induced diabetic obese mice by restoring adiponectin, adiponectin receptors, and APPL1, further improving metabolic homeostasis in the liver and muscle as well as β-cell function. However, 100 mg/kg THC appears to exert beneficial effects in diabetic obese mice through the reduced food intake. These results provide potential mechanisms for THC as an effective therapeutic for treating diabetes in obese subjects.

## Figures and Tables

**Table 1:** Effect of THC on body weight, food intake, relative organ weights and serum parameters in HFD/STZ model C57BL/6J mice.

	ND	HFD/STZ	HFD/STZ + THC 20 mg/kg	HFD/STZ + THC 100 mg/kg	ND + THC 100 mg/kg
Body weight gain and food intake					
Initial body weight (g)	25.94 ± 1.50 ^bc^	28.21 ± 0.63 ^a^	27.38 ± 1.24 ^ab^	28.25 ± 0.71 ^a^	25.11 ± 0.38 ^c^
Final body weight (g)	30.32 ± 1.40 ^bc^	37.49 ± 1.06 ^a^	32.24 ± 1.77 ^b^	32.66 ± 0.52 ^b^	29.67 ± 0.97 ^c^
Body weight gain (g)	4.38 ± 0.37 ^b^	9.28 ± 1.07 ^a^	4.86 ± 1.01 ^b^	4.42 ± 0.66 ^b^	4.60 ± 0.58 ^b^
Food intake (g/mouse/day)	3.39 ± 0.09 ^a^	3.08 ± 0.18 ^c^	3.21 ± 0.05 ^b^	2.92 ± 0.10 ^d^	3.48 ± 0.03 ^a^
Relative organ weights					
Liver (%)	4.11 ± 0.17 ^b^	4.85 ± 0.51 ^a^	4.96 ± 0.40 ^a^	5.36 ± 0.55 ^a^	4.11 ± 0.11 ^b^
Kidney (%)	1.47 ± 0.09 ^a^	1.35 ± 0. 19 ^a^	1.48 ± 0. 15 ^a^	1.38 ± 0.07 ^a^	1.46 ± 0.06 ^a^
Spleen (%)	0.21 ± 0.02 ^b^	0.21 ± 0.03 ^b^	0.32 ± 0.03 ^a^	0.30 ± 0.03 ^a^	0.22 ± 0.04 ^b^
Pancreas (%)	0.56 ± 0.05 ^a^	0.42 ± 0.06 ^b^	0.48 ± 0.04 ^ab^	0.49 ± 0.07 ^ab^	0.54 ± 0.04 ^a^
Fasting serum biochemical parameters					
TG (mg/dL)	61.67 ± 3.73 ^b^	92.00 ± 18.33 ^a^	41.67 ± 10.67 ^c^	40.00 ± 8.94 ^c^	55.00 ± 5.00 ^b,c^
TCHO (mg/dL)	78.75 ± 3.31 ^c^	130.00 ± 7.07 ^a^	95.00 ± 5.00 ^b^	105.00 ±12.58 ^b^	56.67 ± 4.71 ^d^
Cholesterol/HDL-C	1.31 ± 0.03 ^a^	1.38 ± 0.09 ^a^	1.38 ± 0.10 ^a^	1.36 ± 0.08 ^a^	1.30 ± 0.14 ^a^
GOT (U/L)	71.43 ± 8.33 ^bc^	90.00 ± 12.65 ^a^	78.00 ± 7.48 ^ab^	63.33 ± 7.45 ^cd^	56.67 ± 4.71 ^d^
GPT (U/L)	31.43 ± 8.33 ^c^	72.00 ± 7.48 ^a^	48.00 ± 9.80 ^b^	38.00 ± 14.70 ^bc^	27.50 ± 4.33 ^c^

Mice were either fed ND and HFD for 6 weeks and then subjected to STZ treatment to induce diabetes. The diabetic obese mice were further orally administration corn oil, 20 and 100 mg/kg for 14 weeks, respectively. Data are presented as mean ± SEM (*n* = 7–8). Statistical differences (*p* < 0.05) between groups were evaluated by one-way ANOVA with Tukey’s post-hoc test and were labeled as different letters (a, b, c, and d).

## Data Availability

The data presented in this study are available upon request from the corresponding author upon reasonable request.

## Supplementary Materials

The following are available online at https://www.mdpi.com/article/10.3390/nu13124552/s1, Figure S1: Effect of THC on body weight in HFD/STZ treated mice, Figure S2: Effect of THC on fat weight and adipocyte size in HFD/STZ treated mice, Figure S3: Effect of THC intervention on whole-body energy metabolism in HFD/STZ treated mice, Figure S4: Attenuation of macrophage infiltration and polarization in adipose tissue by THC in HFD/STZ treated mice, Figure S5: Upregulation of adipose UCP-1 by THC in HFD/STZ treated mice, Figure S5. THC improved AdipoR-APPL1, insulin signaling, and protein expression involved in lipid metabolism in skeletal muscle of HFD/STZ treated mice.
