# Supplementary material for: Tetrahydrocurcumin Upregulates the Adiponectin-AdipoR Pathway and Improves Insulin Signaling and Pancreatic β-Cell Function in High-Fat Diet/Streptozotocin-Induced Diabetic Obese Mice
Source: Nutrients. 2021 Dec 19;13(12):4552. doi: 10.3390/nu13124552 (PMC8707974; doi:10.3390/nu13124552)
Supplement: Supplementary file 1 [file nutrients-13-04552-s001.zip › nutrients-1462706-supplementary.pdf]

Supplementary Figures: Supplementary Figures S1−S5

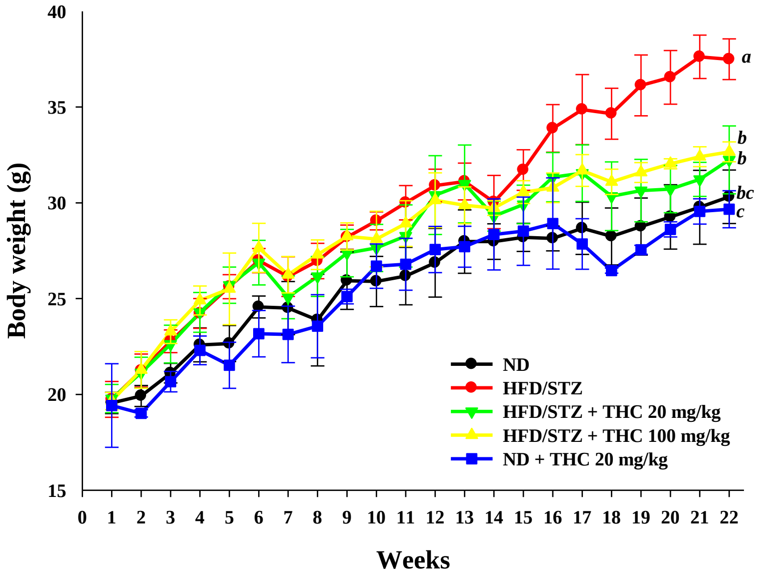

Supplementary Fig. S1. Effect of THC on body weight in HFD/STZ treated mice. Mice were fed an either ND or HFD for 6 weeks and then the mice with HFD fed were i.p. injection of STZ at week 7. After STZ treatment, mice with hyperglycemia was confirmed at week 8 by fasting blood glucose > 200 mg/dl, and subjected to oral 20 and 100 mg/kg THC for 14 weeks with a continued HFD feeding. Change in average body weight during the experiment were recorded (n=7-8). Statistical differences (*P*< 0.05) between groups were evaluated by one-way ANOVA with Tukey's post-hoc test and were labeled as different letters (a, b, and c).

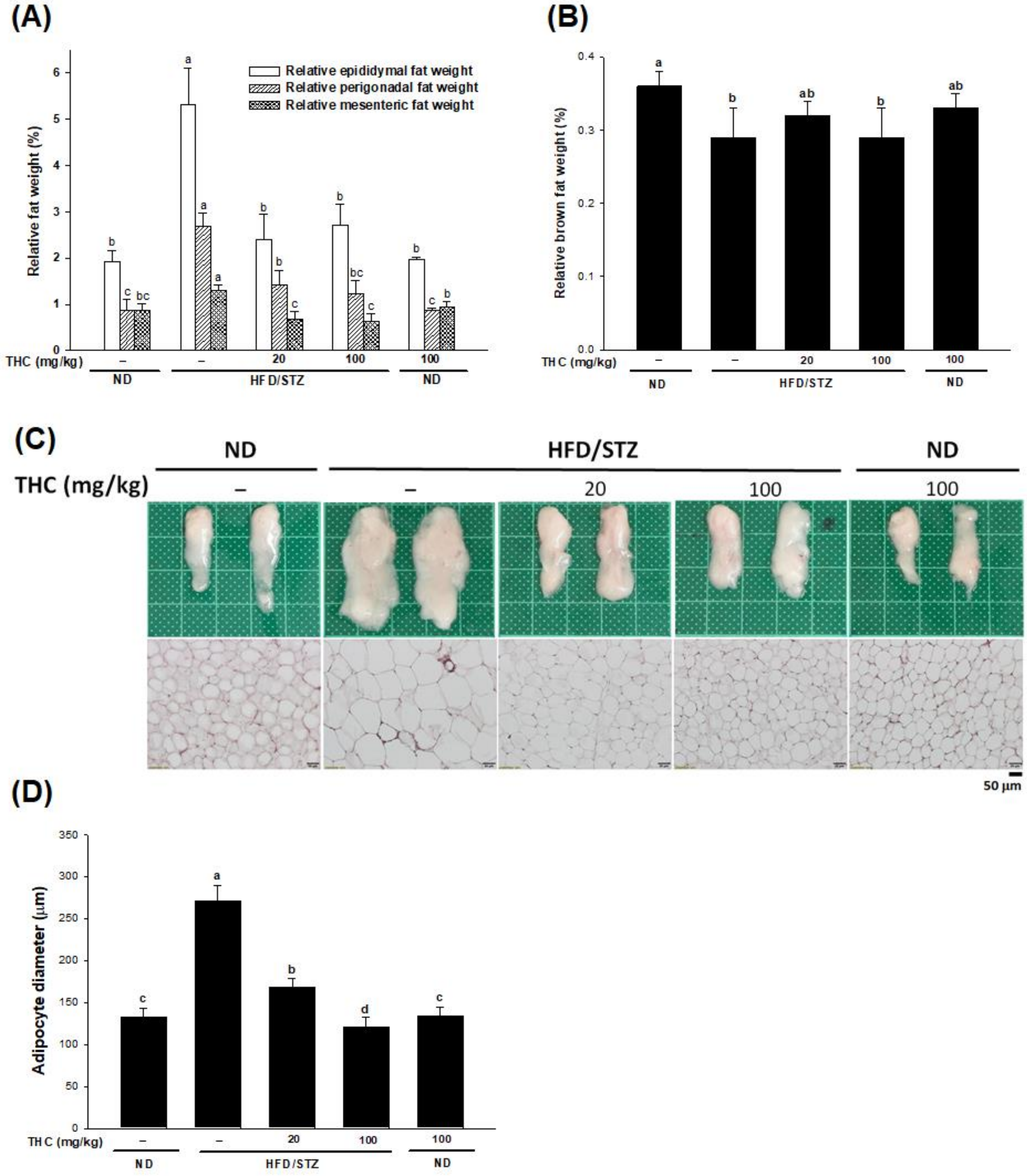

Supplementary Fig. S2. Effect of THC on fat weight and adipocyte size in HFD/STZ treated mice. Relative (A) perigonadal, perirenal, mesenteric, and (B) brown fat weight was expressed as a percentage of body weight (adipose tissue weight/body weight × 100). (C) Representative image and H&E staining of epididymal adipose tissue. (D) Quantitation of adipocyte size in epididymal adipose tissue. Data are presented as mean ± SEM (n=6-8). Statistical differences (*P*< 0.05) between groups were evaluated by one-way ANOVA with Tukey's post-hoc test and were labeled as different letters (a, b, and c).

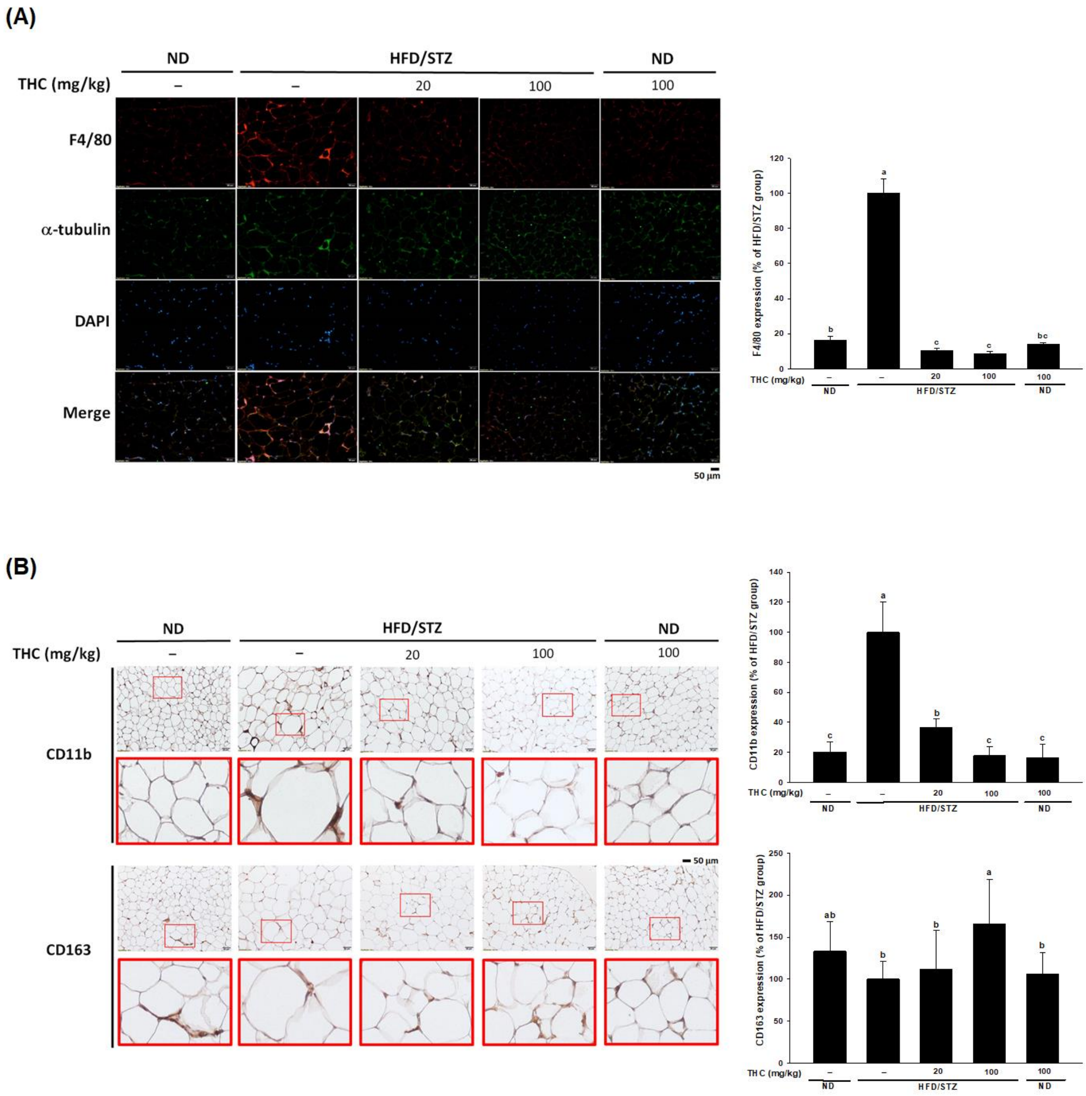

Supplementary Fig. S3. **Attenuation of macrophage infiltration and polarization in adipose tissue by THC in HFD/STZ treated mice.** (A) Macrophage infiltration in epididymal adipose tissue was identified by immunofluorescence staining with hepatic F4/80 (red), α-tubulin (green), and DAPI (blue). (B) Identification of M1 and M2 macrophage using immunohistochemical staining with CD11b and CD163 antibody, respectively. Quantitation of F4/80, CD11b and CD163 was performed by ImageJ (n=5). Statistical differences ($P< 0.05$) between groups were evaluated by one-way ANOVA with Tukey's post-hoc test and were labeled as different letters (a, b, and c).

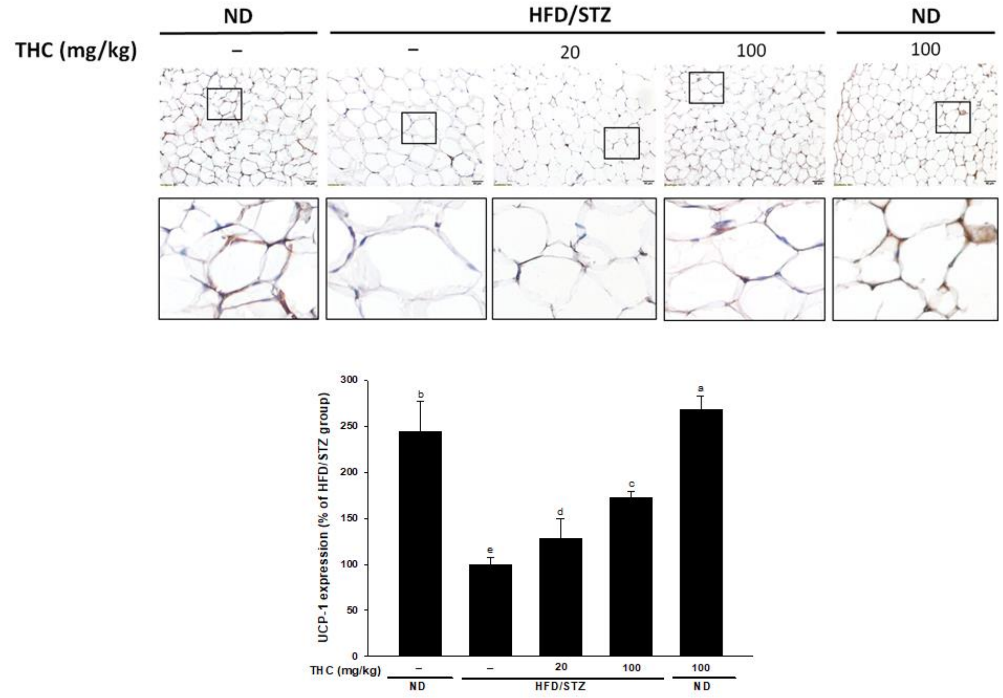

Supplementary Fig. S4. Upregulation of adipose UCP-1 by THC in HFD/STZ treated mice. Immunohistochemical staining and quantitation of UCP-1 in epididymal adipose tissue. Data are presented as mean ± SEM (n=5). Statistical differences (P< 0.05) between groups were evaluated by one-way ANOVA with Tukey's post-hoc test and were labeled as different letters (a, b, and c).

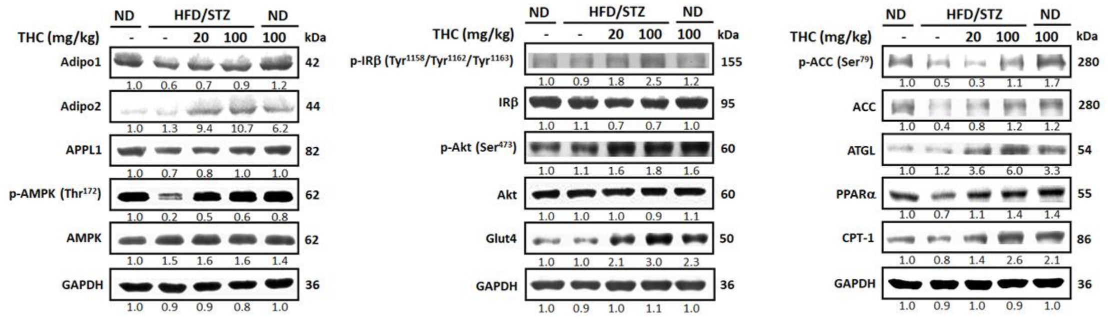

Supplementary Fig. S5. THC improved AdipoR-APPL1, insulin signaling and protein expression involved in lipid metabolism in skeletal muscle of HFD/STZ treated mice. The protein level of AdipoR-APPL1 signaling (left), insulin signaling (middle), lipogenesis and fatty acid oxidation (right) in skeletal muscle was detected by western blot analysis. The results shown are representative of three independent experiments with similar results.
